# Learning from Simple Ebooks, Online Cases or Classroom Teaching When Acquiring Complex Knowledge. A Randomized Controlled Trial in Respiratory Physiology and Pulmonology

**DOI:** 10.1371/journal.pone.0073336

**Published:** 2013-09-09

**Authors:** Bjarne Skjødt Worm

**Affiliations:** Department of Anesthesia and Intensive Care, Copenhagen University Hospital Bispebjerg, Bispebjerg, Denmark; University of Cape Town, South Africa

## Abstract

**Background and Aims:**

E-learning is developing fast because of the rapid increased use of smartphones, tablets and portable computers. We might not think of it as e-learning, but today many new e-books are in fact very complex electronic teaching platforms. It is generally accepted that e-learning is as effective as classroom teaching methods, but little is known about its value in relaying contents of different levels of complexity to students. We set out to investigate e-learning effects on simple recall and complex problem-solving compared to classroom teaching.

**Methods:**

63 nurses specializing in anesthesiology were evenly randomized into three groups. They were given internet-based knowledge tests before and after attending a teaching module about respiratory physiology and pulmonology. The three groups was either an e-learning group with eBook teaching material, an e-learning group with case-based teaching or a group with face-to-face case-based classroom teaching. After the module the students were required to answer a post-test. Time spent and the number of logged into the system was also measured.

**Results:**

For simple recall, all methods were equally effective. For problem-solving, the eCase group achieved a comparable knowledge level to classroom teaching, while textbook learning was inferior to both (p<0.01). The textbook group also spent the least amount of time on acquiring knowledge (33 minutes, p<0.001), while the eCase group spent significantly more time on the subject (53 minutes, p<0.001) and logged into the system significantly more (2.8 vs 1.6, p<0.001).

**Conclusions:**

E-learning based cases are an effective tool for teaching complex knowledge and problem-solving ability, but future studies using higher-level e-learning are encouraged.Simple recall skills, however, do not require any particular learning method.

## Introduction

Historically, many learning methods have been used, but in recent years e-learning has been increasingly integrated into medical education with the expansion and dissemination of digital platforms for everyday use [1]. These educational applications are being developed for both pre- and postgraduate training (examples are: eFront, Moodle, Dokeos, Claroline, Ilias etc.) and used at Universities as part of their curriculum. E-learning differs from former educational methods in the shift from teaching to learning, in which the student is required to actively search knowledge instead of being a passive recipient of such [2]. E-learning is developing fast because of the rapid increased use of smartphones, tablets and portable computers. We might not think of it as e-learning, but today many new e-books are in fact very complex electronic teaching platforms with videos and complex animations. Because of this development a new systematic description of e-learning content is needed [1], [3]–[5]. A recent contender for a useful taxonomy may be found at the Upsidelearning-website [6]. When using this taxonomy, e-learning may be divided into tree different *types* and three different *levels*. *Type* corresponds to the learning method and are divided into 1. Presentations (simple e-learning with no interactivity), 2. Scenarios (interactions fx cases that allow the learner to take decisions) or 3. Games/simulations (complex computer based patient scenarios with multiple students interacting). *Level* refers to the multimedia development level. Multimedia *level 1* includes text, basic images, audio, simple interactivities for content presentation and a template layout used trough all e-learning pages. *Level 2* adds video, simple animations and variations on the presented e-learning pages. *Level 3 have* complex animation, high fidelity/3D graphics, complex multilevel and multivariable interaction. Studies in e-learning materials may thus be divided into nine different categories. Furthermore, non-e-learning methods may also be divided into the same three *types* of teaching (presentations, scenarios, and simulations). Learning objectives include recall, analysis and problem solving, each of which may be achieved to different degrees for each learning type and level [7]–[10]. Comparing the same type of learning (only changing the method) will then primarily show effects depending on teaching method.

The multimedia-level could be of importance when comparing different interventions. Therefore one could theorize that different outcome from one type of e-learning to another could be from differences in multimedia-level alone.

A major issue when discussing the value of e-learning is the potential difference in material quality, communication skills and digital setup, making comparisons between learning methods uneven and hard to quantify. This highly variable description methodology used when describing e-learning results in lacking reliability of data and thereby research describing the efficacy of e-learning. The quality of many studies is also questionable: control groups are either lacking or not well defined [11].

Questioning the effects of e-learning versus no intervention is no longer relevant [12]. in fact many studies have shown an effect similar in quality to traditional classroom teaching [11]. However, this is not adequate when discussing the true place of medical e-learning since learning material widely differ between studies. Little is known about e-learning's value in relaying contents of different levels of complexity to students. We set out to investigate e-learning effects on simple recall and complex problem-solving compared to classroom teaching.

### Aim of the study

#### Type of learning

Since previous studies are highly variably and typically compare mixed *levels* of learning methods or don't use relevant control groups, our aim was to compare two different *types* of e-learning (e-book and e-cases) with traditional scenario-based teaching with the same *level* of learning (level 1), either with a simple or a complex learning *content*. We didn't use video or animations, and all pages were standard view. This method is often used when teaching senior medical students in Denmark. We reasoned that if simple level-1 e-learning was comparable to classroom teaching, then higher levels would be at least as good, if not better.

The learning objective of simple content included recall of lung volume curve, while the learning objective of complex content included advanced pulmonology cases where the student was required to use complex knowledge involving both knowledge about respiratory physiology and pulmonology.

## Materials and Methods

### Ethics Statement

The study was purely educational and the Danish National Committee on Health Research Ethics (DNVK), Regional Region was consulted. Their conclusion was that study did not require ethical approval (h-4–2013-fsp 41).

### Learning courses and groups

Test subjects were Danish nurses on their first course specializing in anesthesiology.

A basic educational course was repeated three times with 21 students participating in each course. Before starting this course, students were given two weeks to take a pre-test of basic points that were to be included in the course itself. No students were admitted to the course without prior completion of the pre-test. The result of the pre-test did not influence participation in the course. Students were randomly divided into three equal numbered groups using computer block randomization. Group 1a (eBook, n = 21) was presented with textbook material electronically, while group 1b (eCase, n = 21) participated in an interactive case-based e-learning program. Group 2 (classroom teaching, n = 21) received case-based classroom education. The subject was ‘the lung volume curve’ and cases relating to both this and pulmonology. In Group 1b and 2 education was based on four case-stories and lung volume curve relevance shown using those. Group 1 was free to use the e-learning module as much as they desired during a two-week span. All students were required to hand in a post-test within two-weeks. The improvement of each participant (post-test result minus pre-test result) was used as statistic for comparison between groups. The amount of time that each student spent on each educational element was also measured.

### Module setup

The pre-test, post-test, questionnaire and e-learning modules were all designed using Moodle - a Free-software (GPLv3 licenced). PHP web application for producing modular internet-based courses integrated into www.medviden.dk, a free Danish homepage for medical education. All parts of the study were closed and required password for admittance, but in the future the e-learning modules will be opened for free access. The program was carried out in Danish.

### Pre- and post-tests

Pre- and post-tests both consisted of 25 questions (10 recall and 15 complex) randomly chosen from a pool of 50 different questions. In order to avoid confounding, the questions were shuffled for every course. The three groups would thereby use all questions in the pool either at pre- or posttest.

The questions were provided in several formats including multiple choice (single best answer from multiple answers) and true/false questions. The questions included clinical photos, and both tests were based on clinical stories. The questions were all used in former examinations at fifth year at Copenhagen University Faculty of Medicine thereby also verified by leading physicians. Both tests were online reviewed (pilot-tested) and validated by two senior doctors working with extensive experience in Pulmonology in Denmark. The questions were rated as being of similar levels of difficulty and of similar clinical relevance, and they ensured that the material covered by the tests was addressed by both the e-learning modules and the keynote presentation. In order to avoid the risk of teaching to the test we created the test-questions after the learning material. The students had a time limit of 40 minutes for completion of each test. Only one correct answer was permitted for each question.

### eBook (group 1a)

The eBook was an ordinary homepage presenting textbook material electronically, excluding clinical cases, pictures or explanations. We did not want to create a bias regarding questioning, and therefore we did not use questions within the eTextbook modules.

### eCases (group 1b)

The eCases module was prepared using clinical cases, pictures and explanations. The same keynote presentation used in Group 2 was uploaded, and the students had the option of reading the slides more than once. Two case-stories were presented, and the student was able to follow more than one path toward the conclusion of the case. We didn't want to create a bias regarding questioning, and therefore we did not use questions within the e-learning modules.

### Classroom teaching (group 2)

The case-based group was presented with a keynote classroom lecture and didactic teaching about the subject. The didactic teaching was prepared with a strict timed manuscript (recall 15 min, cases 30 min) and followed one path trough each case. Two case-stories were presented and discussed within the group. The teacher was available for questioning.

### Time-measurements

All students were required to be present during the entire session of the classroom teaching. Both e-learning modules used course login-times measured within Moodle.

### Questionnaire

All participants were asked to complete a questionnaire after completing the post-test. The questionnaire obtained general feedback regarding the educational method. A PDF version of the questionnaire is available [in Danish] upon request.

### Study size and statistical tests

Deriving experiences from a pilot study, the posttest-pretest difference of positive answers in group 2 (eBook) was anticipated to be 30% higher (an improvement in correct answers from 30% to 60%). Anticipated range for this difference would be 20–40%, thereby applying a standard deviation for all groups at 5%. We expected that all educational methods would only deviate slightly from each other, and a 5% difference was chosen as a MIREDIF. Significance level was set at 5%, and statistical power at 80%. This yielded a total of 16 subjects in each group [http://www.opengcp.dk/calmiredif.php]. To avoid an impact due to dropouts or missing data, it was decided that each group consisted of 21 subjects. Posttest-pretest difference for each participant was chosen as our primary statistic. Given the limited number of participants, the Mann-Whitney U-test was chosen for between-group comparisons with an ANOVA as an initial omnibus test

### Outcomes

Primary outcome was comparisons between improvements in the three groups.

## Results

All 63 students concluded both pre- and post-tests and the educational elements of the study. Pre-test and post-test results are presented in table 1, with between-group comparisons in table 2 and 3. The three groups have comparable pre-test results (table 3). For recall of simple knowledge (the lung volume curve), all educational methods improved knowledge almost to maximum and were equally successful (table 2). For analysis of complex scenarios (pulmonology cases), the group given textbook material (group 1a) acquired significantly less improvement than either of the other two groups (eCase or Classroom) (table 2). Time spent with educational material was significantly less in the textbook group (p<0.001) and significantly more in the eCase group compared to the classroom learning group (p<0.001, Table 4). Group 1b had significantly (p<0.001) more logins (2.8) at the web-application as did group 1a (1.6) (table 4).

**Table 1:** Correct answers in pre-tests and post-tests.

Group	Pre-test	Post-test	Improvement	Learning efficiency
1a (eBook), Simple	27.4% (25.4–29.4%)	92.9% (90.6–95.2%)	65.5% (63.0–67.9%)	0.89 (0.85–0.94)
1b (eCase), Simple	26.7% (24.7–28.6%)	94.3% (92.2–96.4%)	67.6% (64.7–70.5%)	0.88 (0.85–0.92)
2 (Case), Simple	27.6% (25.9–29.4%)	93.3% (91.4–95.3%)	65.7% (63.0–68.4%)	0.88 (0.84–0.92)
1a (eBook), Complex	33.7% (32.5–34.9%)	82.1% (77.9–86.3%)	48.4% (44.1–52.8%)	0.92 (0.76–1.1)
1b (eCase), Complex	33.3% (32.1–34.5%)	92.7% (91.2–94.2%)	59.4% (57.6–61.1%)	0.51 (0.45–0.56)
2 (Classroom), Complex	33.0% (31.6–34.4%)	91.9% (90.3–93.5%)	58.9% (56.8–61.0%)	0.59 (0.57–0.61)

Values are relative numbers of correct answers for each type of educational content in each group, with 95% confidence intervals in brackets. Learning efficiency is improvement in correct questions per minute of study. N = 21 in each group.

**Table 2:** Between-group comparisons in test result improvements.

*Recall of simple knowledge (Lung volume curve)*		
ANOVA	f	p-value
eBook, eCase, Classroom	0.73	0.49

*Analysis of complex scenarios (Pulmonology cases including physiology)*			
ANOVA	f		p-value
eBook, eCase, Classroom	16.77		<0.001
Mann-Whitney U-test	Results	Difference	p-value
1 (eBook) vs 1b (eCase)	48.4% vs 59.4%	3.3 (1.7–4.8)	<0.001
1 (eBook) vs 2 (Classroom)	48.4% vs 58.9%	3.1 (1.7–4.6)	<0.001
1b (eCase) vs 2 (Classroom)	59.4% vs 58.9%	0.1 (−0.7–1.0)	0.58

Results in percent, difference in correct answers. N = 21 in each group.

**Table 3:** Between group comparisons in average time spent on educational material by students in each group, for simple and complex problems.

*Recall of simple knowledge (Lung volume curve)*			
ANOVA	f		p-value
eBook, eCase, Classroom	2.98		0.059

*Analysis of complex scenarios (Pulmonology cases including physiology)*			
ANOVA	f		p-value
eBook, eCase, Classroom	42.73		<0.001
Mann-Whitney U-test	Results	Difference	p-value
1 (eBook) vs 1b (eCase)	18 (15–20) vs 38 (33–42)	20 (14–25)	<0.001
1 (eBook) vs 2 (Classroom)	18 (15–20) vs 30 (29–31)	12 (9–15)	<0.001
1b (eCase) vs 2 (Classroom)	38 (33–42) vs 30 (29–31)	8 (4–12)	0.002

Time spent in minutes, with 95% confidence interval in brackets. N = 21 in each group.

**Table 4:** Average times group 1a and group 1b were logged in to the web-application.

Group comparisons	Results	P-value
1 (eBook) vs 1 (eCase) [times]	1.6 (1.3–1.9) vs 2.8 (2.4–3.1)	<0.001

Times logged in, 95% confidence interval in brackets. Mann-Whitney U-test for comparisons. N = 21 in each group.

## Discussion

### Benefit of learning methods (types)

There can be no doubt that e-learning works. All students managed to improve in their post-test scores compared to the pre-test scores. This was as we expected. Also the differences between learning types (overall) were anticipated but what is more interesting is the disparity between complex and simple learning differences.

The improvement in the recall and learning efficiency of simple knowledge was equal in all groups, while group 1a (eBook) was significantly less effective in the recall of complex knowledge but had higher learning efficiency. This was to be expected because of the longer learning times in both classroom teaching and e-cases.

We can conclude that the recall of simple knowledge does not seem to be dependent on the learning method, while on the other hand analysis and problem-solving seems to be dependent on a case-based method, which may be implemented equally well by eCases or classroom teaching. The lower learning efficiency is noteworthy, but combined with the lower outcome it is probably not preferable.

It is also noteworthy that level 1 eCases achieves the same good results as face-to-face case-based teaching, meaning that even complex knowledge can be taught with simple (multimedia level 1) e-learning. We believe that some of the traditional teaching could be delivered as e-learning without loss of quality and over time this would be a cheaper solution. This teaching method is also interesting because of the ability to try the cases again. This finding supports the thesis made by Cook [13], but that it is better than other learning methods is notable. Group 1b was logged in 2.8 times (table 4) and spent significantly longer time with the case training as did both the classroom group and the eBook group.

It is also interesting to note that students spent less time delving on the eBooks, which generated the poorest result for the complex scenarios, suggesting that the students either overestimated the breadth of the knowledge they attained from the eBooks, that it is hard for students to transform this theoretical text into complex problem-solving patient cases or that eBooks are actually better in the beginning, but that the students will lack some aspects. If the last is true it will support the findings from others [14]–[16] who argue that eBooks should be integrated in the curriculum, but only as an adjunct.

### The right e-learning type and level for the task in mind

It is important to choose the right educational method to the content and purpose of the educational material. E-learning has some benefits while traditional teaching has others.

Unfortunately e-learning-platforms have not yet developed to accommodate the social aspect of traditional classroom teaching [4], [5]. Much of this is being developed as we speak as is social networks like Facebook, Google plus etc. That being said e-learning has potential advantages over didactic learning, both when looking at accessibility and advanced contents (multimedia and interactive navigation). This study has investigated simple e-learning tools, but we hypothesize that higher multimedia levels of e-learning may lead to competitions and direct student interactions which may eventually resemble those seen in a classroom setting [1]. At the end of the day, however, it may all come down to cost-benefit analyses. How are we able to create the best learning environment using minimal resources? When the conclusion is that e-learning is as good as traditional teaching, this could be a key argument for such a way of thinking [10]. Our study does not compare development costs versus learning potential and this kind of study would probably be very hard to repeat. We do theorize that higher levels and types of e-learning would be more expensive than both didactic teaching and lower-level e-learning. As of now we are still unable to determine if there are major benefits for higher-level of e-learning compared to lower-level of e-learning, or which levels of e-learning may adequately improve higher-order skills such as cognitive abilities in the students [17]. From the students perspective it is important to remember that this way of arguing is from a teaching perspective. As we have seen it is not necessarily faster to learn this way, it just yields a higher outcome.

### E-learning in the future

In his review from 2004 Wutoh concluded that there is no significant difference between e-learning and didactic medical teaching [18]. Since then there has been a tremendous development of both software (e-learning contents and levels) and hardware (mobile devises etc.) suggesting an even better effect today. Both simple and complex knowledge can be taught and even though other skills are not as easy to teach using e-learning (readily available knowledge, manual dexterity, clinical experience and cognitive abilities) future technological developments catering to such complex skill sets may not be far away with the increasing use of computer games, virtual reality simulations and social networks [7].

### Limitations

There are a number of potential limitation to our study. One is the theoretical risk of teaching-to-the-test bias, especially in group 2 (classroom teaching), but we believe this bias has been reduced by the creation of the tests *after* creating teaching materials, In addition, the direction of such a potential bias would reduce the improvements seen after case-based learning, which would improve the difference between group 1b and group 2. Nevertheless, we can confirm that case-based learning methods are superior (at improvement), when teaching advanced knowledge and problem-solving ability, and that eCase is at least as good as classical, case-based face-to-face teaching [17].

Validity of pre-test – post-test scores is also discussed, but in our case the questions were randomized thereby minimizing the risk. The time measured inside the module may not be precise, but the students were auto-logged- out after 5 minutes without activity in order to obtain the most accurate values. The teacher did the time measuring in “classroom teaching” thereby applying theoretical risk of bias.

## Conclusions

The results of this study promote E-learning – not because it is better than face-to-face teaching when looking at the results, but because the outcome is the same and it provides the students with a lot of possibilities. We can also conclude that e-learning with cases works significantly better than e-learning with only textbook material (at the same level) when learning complex knowledge and looking at knowledge gain. Simple knowledge can be learned equally well with all learning methods.

We suggest that future studies should evaluate whether different multimedia levels of e-learning could in fact provide better results. We hope that the variable description methodology formerly used can be standardized and that the focus will no longer be on whether e-learning works, but what should be taught using this method of teaching.
